# Development of a High-Performance Composite Mortar for Ultra-High-Strength Preplaced Aggregate Concrete-Filled Steel Tubes (PACFSTs)

**DOI:** 10.3390/ma18102218

**Published:** 2025-05-11

**Authors:** Yicheng Zhao, Xiaojun Zhou, Yingda Zhang, Sheng Li

**Affiliations:** 1 Department of Road and Bridge Engineering, Sichuan Vocational and Technical College of Communications, Chengdu 610130, China; 2 School of Architecture and Civil Engineering, Xihua University, Chengdu 610039, China

**Keywords:** high-performance composite mortar, sand-to-binder ratio, water-to-binder ratio, preplaced aggregate concrete-filled steel tube, volumetric stability, expansive agent

## Abstract

This study developed a high-performance composite mortar with a low water-to-binder (W/B) ratio to improve the mechanical strength and volumetric stability of preplaced aggregate concrete-filled steel tubes (PACFST). Silica fume was incorporated to optimize the interfacial transition zone (ITZ) between the matrix and coarse aggregates. The effects of the sand-to-binder (S/B) ratio, water-to-binder (W/B) ratio, and expansive agent content on the flowability, compressive strength, and volume stability of the composite mortar were systematically analyzed. Experimental tests were conducted using vibration-free molded specimens, and the influence of different S/B ratios (0.8–1.4), W/B ratios (0.26–0.32), and expansive agent dosages (0–8%) on mortar properties was evaluated. The results indicate that an optimal S/B ratio of 1.2 significantly enhances flowability and strength, whereas further increases offer limited improvement. Reducing the W/B ratio enhances strength, with a decrease from 0.32 to 0.28 leading to a 23.4% increase in 28-day compressive strength. Additionally, a 6% expansive agent dosage reduces 90-day shrinkage by 13.1% while maintaining high compressive strength. The optimized PAC achieved a 28-day compressive strength of 115.9 MPa, with an 11.6% increase in 7-day strength and a 51.2% reduction in 90-day shrinkage compared to conventional C100 concrete. These findings provide theoretical guidance for designing high-strength, low-shrinkage PAC, offering insights for bridge, tunnel, and high-rise building applications.

## 1. Introduction

Preplaced aggregate concrete-filled steel tubes (PACFSTs) have been widely applied in major engineering projects such as bridges, tunnels, and high-rise buildings, owing to their high load-bearing capacity, excellent seismic performance, and superior durability [1,2]. Recent studies demonstrate that the interfacial transition zone (ITZ) between the grout and aggregates critically governs PACFST performance [3,4,5], but traditional grouts often exhibit weak ITZ bonding and excessive shrinkage [6]. Advanced ITZ modification strategies, such as silica fume densification [7] and nano-CaCO_3_ incorporation [8], have shown promise in conventional concrete but remain underexplored for PACFSTs. Moreover, shrinkage compensation via MgO/CaO-based expansive agents requires optimization for high-strength mortars [9].

In the PACFST construction process, coarse aggregates are first filled into the inner cavity of the steel tube, followed by the injection of cement grout. This necessitates that the grout possesses excellent fluidity, high strength, and low volumetric shrinkage to ensure adequate filling of the coarse aggregates and the formation of a high-quality interfacial transition zone (ITZ) [10,11]. However, the application of traditional cement grouts in PACFST structures still faces challenges such as weakened ITZ, significant volumetric shrinkage, and slow early strength development. Therefore, there is an urgent need to optimize the grout mixture to enhance the overall performance of PACFST [12].

PACFSTs have received relatively little research attention thus far. However, based on studies of conventional concrete-filled steel tubes (CFSTs), it is well-established that the performance of the core concrete significantly influences the overall behavior of the CFST component [13]. Hou et al. [14] found that the presence of core concrete markedly improves the shear resistance of CFST columns. Similarly, Sakino et al. [15] discovered that the strength of the concrete material has a profound impact on the performance of CFSTs. Therefore, when the quality of the steel tube material is stable, the factors affecting the performance of PAC can be inferred from investigations into the factors influencing the performance of preplaced aggregate core concrete [16].

Preplaced aggregate concrete (PAC) exhibits lower interfacial bond strength between the grout and coarse aggregates compared to conventional concrete, making it difficult to effectively restrain the relative displacement and rotation of the coarse aggregates under axial pressure, which results in the lower compressive strength of PAC [17,18]. Therefore, to enhance the strength grade of PAC, it is necessary to improve the ITZ between the grout and coarse aggregates, thereby strengthening the adhesive force between the materials. Ayanlere et al. [19] suggested that the water-to-cement ratio has a significant impact on the strength of PAC, and when the water-to-binder ratio is relatively high, the influence of the cement-to-sand ratio is less pronounced. They also found that the concrete strength increases with the addition of expansive agents, contributing significantly to the strength improvement of confined PAC. Duan et al. [20] believed that the incorporation of silica fume into cement reduces the average size of Ca(OH)_2_ crystals in the ITZ. The SiO_2_ in silica fume can react with some of the Ca(OH)_2_ to form C–S–H gel, which increases the density, improves the interface structure, and enhances the overall performance of the concrete [21]. The microhardness of the interface region is significantly improved after the addition of 5% silica fume. To improve the ITZ of PAC and enhance the overall performance of PACFSTs, this study primarily focused on reducing the water-to-binder ratio of the composite mortar to increase its strength, while ensuring adequate fluidity. This is supplemented by the addition of an appropriate amount of expansive agent to compensate for shrinkage. The goal is to develop a composite mortar grouting material with high fluidity, good stability, low volumetric shrinkage, and ultra-high strength, with the aim of producing ultra-high-strength preplaced Aggregate concrete-filled steel tubes.

Volumetric stability remains a critical challenge in PACFST systems due to autogenous and drying shrinkage of the grout [22]. Expansive agents, particularly CaO- and MgO-based compounds, have demonstrated efficacy in compensating for shrinkage through controlled hydration reactions [23]. When incorporated into cementitious matrices, these agents generate brucite (Mg(OH)_2_) or portlandite (Ca(OH)_2_), inducing micro-expansion that counteracts shrinkage stresses [24]. Recent advances highlight that optimal dosages (4–8%) can reduce 90-day shrinkage by 15–30% without compromising strength in conventional concrete [25]. However, their application in high-strength PACFST grouts with a low W/B ratio remains underexplored, especially regarding compatibility with mineral admixtures such as silica fume.

High-performance composite mortar (HPCM) can effectively improve the rheological properties, strength, and shrinkage characteristics of the grout by optimizing the water-to-binder ratio (W/B), the sand-to-binder ratio (S/B), and the proportion of admixtures and expansive agents [26,27]. Studies have shown that a low W/B and a high S/B can enhance the density and strength of the ITZ, but an excessively high S/B may lead to a decrease in grout fluidity, affecting construction performance [28]. Furthermore, the appropriate addition of expansive agents can compensate for the autogenous and drying shrinkage of the grout, improving the volumetric stability of the PAC system [29,30]. Therefore, an in-depth investigation into the effects of the sand-to-binder ratio, the water-to-binder ratio, and the amount of expansive agent on the performance of HPCM is of great significance for the optimization of PAC structures.

While the PAC technology is well-established, its application in steel tubes faces unresolved challenges: (1) weak interfacial transition zones (ITZ) due to poor grout–aggregate bonding [31] and (2) excessive shrinkage in systems with a low water-to-binder ratio (W/B) [32]. The existing studies have predominantly focused on conventional concrete or high-W/B grouts, leaving a critical gap for ultra-high-strength PACFSTs with volumetric stability.

This study, based on non-vibratory molding experiments, investigated the influence of the sand-to-binder ratio (0.8–1.4), water-to-binder ratio (0.26–0.32), and CaO–MgO expansive agent content (0–8%) on the fluidity, compressive strength, and volumetric stability of the composite mortar. Furthermore, it evaluated the enhancing effect of optimized mix proportions on the mechanical properties of PAC. The research findings can provide a scientific basis for material optimization in the PAC system and offer technical guidance for the engineering application of high-strength, low-shrinkage PAC.

## 2. Experimental Program

### 2.1. Materials

The cement used in the composite mortar for PACFSTs was P·O 42.5, while P·O 52.5 cement was used for conventional C100 CFST. The technical indicators of the cement are shown in Table 1. Fly ash cenospheres with an aluminosilicate composition (SiO_2_, ~55%; Al_2_O_3_, ~30%) were used, exhibiting a specific surface area of 1300 m^2^/kg and a water demand ratio of 90%. Fly ash cenospheres were incorporated as a multifunctional additive to improve workability as their spherical morphology reduces interparticle friction, enhancing grout flowability at low water-to-binder ratios [33]. Moreover, as rigid inclusions, they restrict matrix deformation during hydration, reducing crack initiation [34]. Silica fume contained 95% SiO_2_ and had a water demand ratio of 122%, measured according to ASTM C1240-20 [35]. The coarse aggregates for preplacing were all crushed pebbles, with a gradation of 10–16 mm:16–25 mm = 3:7 and a vibrated void ratio of 34.7%. For the C100 concrete, 5–16 mm basalt crushed gravel was used. The manufactured sand had a fineness modulus of 2.8 and contained 6% stone powder. Admixtures, including polycarboxylate water-reducing admixtures (WRAs) and bleeding inhibitors, were provided by Sanrui Polymer Materials Co., Ltd. (Taizhou, China). The expansive agent, provided by Subote New Materials Co., Ltd. (Nanjing, China), was a CaO–MgO-based compound (technical indicators provided in Table 2), designed to compensate for shrinkage through hydration-induced expansion without aluminum-based reactions. Tap water from the laboratory was used.

### 2.2. Experimental Test

**Figure 1:**
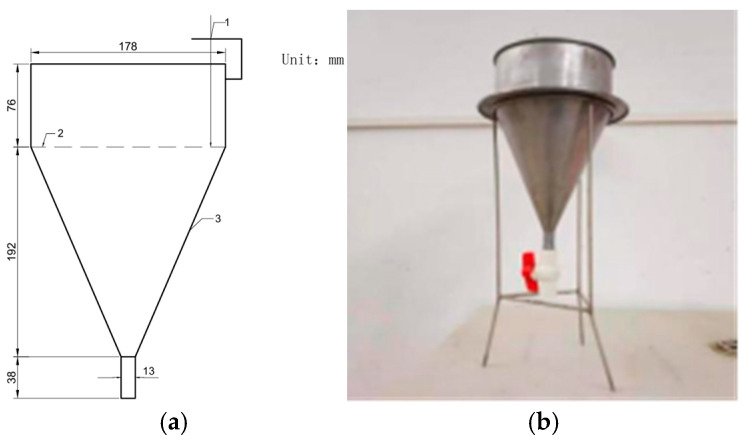
(**a**) Schematic diagram of the flow cone (dimensions labeled). (**b**) Photograph of the physical flow cone apparatus.

The mortar fluidity test method followed the procedure for determining the fluidity of cement paste as outlined in the Specifications for Cement and Concrete Tests in Highway Engineering (JTGE30 [36]), specifically test method T0508. The dimensions and appearance of the fluidity cone are shown in Figure 1. For the mechanical performance test methods, the compressive strength of the grout material was tested according to the Method of Testing Cements—Determination of Strength (ISO method) (GB/T 17671 [37]). The compressive strength and elastic modulus of preplaced aggregate concrete-filled steel tubes were tested following the Standard Test Methods for Mechanical Properties of Concrete (GB/T50081 [38]). Circular steel tubes with nominal dimensions of 150 mm outer diameter and 5 mm wall thickness, filled with preplaced aggregate concrete, underwent 28 days of standard moist curing (20 ± 2 °C, RH ≥ 95%) prior to testing. Paste stability was assessed through direct visual observation for (1) the formation of a free water layer (bleeding) and (2) non-uniform distribution of aggregates (segregation). Fresh mixtures were maintained in sealed transparent containers at 25 ± 2 °C and examined at 15 min intervals over 2 h. Stability was confirmed when no visible phase separation or settlement occurred throughout the observation period. The volume stability test method for preplaced aggregate concrete was conducted using the contact method specified in the Standard for Test Methods of Long-Term Performance and Durability of Ordinary Concrete (GB/T 50082 [39]).

### 2.3. Mix Design

As the grouting material for preplaced aggregate concrete-filled steel tubes, the composite mortar needs to exhibit high fluidity, excellent volume stability, low shrinkage, and high strength. Previous research has shown that a single-doped fly ash system adversely affects the early strength of the mortar and fails to meet the requirement for one-time vibration-free application in terms of fluidity. Therefore, the experiment adopted a dual-doping system with microspheres and silica fume to further leverage the filling effect, pozzolanic effect, and micro-aggregate effect of mineral admixtures. Through experimentation, a microsphere content of 20% and a silica fume content of 8% were selected, with a water-to-binder ratio of 1.0. The S/B ratios (0.8–1.4) and water contents were selected based on preliminary trials. Mortars with S/B < 0.8 exhibited poor cohesion, while S/B > 1.4 caused segregation. For each S/B, the water content was tuned to achieve a target flow time of 25–40 s.

The experiment was designed to adjust the sand-to-binder ratio in order to optimize the workability, mechanical properties, and shrinkage performance of the composite mortar. Modification of the sand-to-binder ratio was expected to improve the volumetric shrinkage and viscosity of the composite mortar, enhance its workability and strength, and enhance its stability and filling performance. In this study, non-vibratory molded specimens were used, and the mix proportions are presented in Table 3.

Excessive water-to-binder ratio is detrimental to the strength and shrinkage of the composite mortar. On the other hand, if the water-to-binder ratio is too low, the viscosity of the cementitious mixture becomes excessively high, making it difficult to ensure dense pouring of concrete-filled steel tubes with preplaced aggregates. However, a lower water-to-binder ratio generally results in higher paste strength. To optimize the performance of the composite mortar, a series of experiments were designed to adjust the water-to-binder ratio. Since the amount of water directly affects the unit weight of the composite mortar, the mix proportions were adjusted based on the measured unit weight of the specimens after molding, ensuring that the error between the designed and measured unit weights was within 2%. The actual mix proportions are shown in Table 4.

Moreover, the experiment was designed to optimize the performance indicators of the composite mortar by incorporating different amounts of the expansive agent into the composite admixture using the external addition method. The mix proportions are presented in Table 5.

## 3. Test Results and Discussion

### 3.1. Influence of the Sand-to-Binder Ratio

#### 3.1.1. Workability

Workability is a crucial indicator for evaluating the constructability of the composite mortar, encompassing primarily fluidity and stability. Fluidity determines the penetration ability of the grout within the preplaced aggregates, while stability influences the uniformity of the grout before setting and the risk of segregation. In this study, the workability of the composite mortar was assessed through spread diameter and flow time measurements, with an analysis of the effects of W/B, S/B, and expansive agent content.

**Figure 2:**
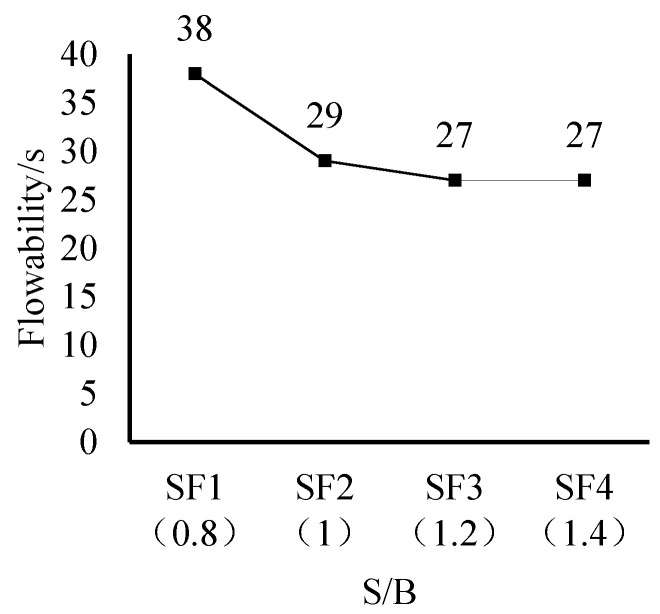
Effect of the sand-to-binder ratio on the properties of the composite mortar.

The experimental results indicate that as the W/B ratio increases, the fluidity of the grout enhances, but an excessively high W/B ratio may lead to bleeding and segregation. The spread diameter of the composite mortar decreased from 285 mm to 220 mm as the S/B ratio increased from 0.8 to 1.2 (Figure 2). At S/B = 1.2, the optimal balance between fluidity (spread diameter: 240 ± 5 mm) and stability was achieved. The optimal flow time (27 s at S/B = 1.2) represents a 35% improvement over high-strength PAC grouts (typically 35–40 s) reported by [40], while maintaining stability—a critical advance for vibration-free applications. Further increases to S/B = 1.4 resulted in marginal changes (spread diameter: 235 ± 5 mm), indicating a threshold effect [41].

**Figure 3:**
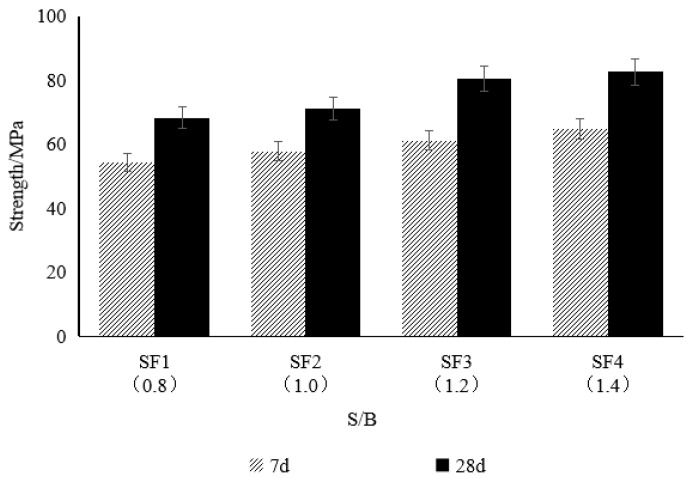
Influence of the sand-to-binder ratio on mortar strength.

As shown in Figure 3, when the S/B ratio increased from 0.8 to 1.2, the spread diameter decreased by 30%, achieving optimal fluidity. When the S/B ratio further increased to 1.4, the fluidity of the composite mortar remained essentially unchanged. This demonstrates that the improvement in the workability of the composite mortar is limited with an increase in the S/B ratio, and the effect is more pronounced within a smaller range of S/B ratios. Additionally, the content of the expansive agent affects the workability of the grout. When the expansive agent content increased from 0% to 6%, the spread diameter slightly decreased, but the change in flow time was insignificant. An appropriate amount of expansive agent can enhance the cohesion of the grout, reduce bleeding, and improve stability. When the content exceeded 6%, the viscosity of the grout significantly increased, leading to a decrease in fluidity and affecting construction adaptability. Based on a comprehensive analysis, optimal workability can be achieved under the conditions of W/B = 0.28, S/B = 1.2, and an expansive agent content of 4–6%. Under these conditions, the grout exhibits excellent fluidity, stability, and resistance to segregation.

#### 3.1.2. Mechanical Properties

As shown in Figure 3, the 7-day and 28-day compressive strengths of the composite mortar increased with an increase in the sand-to-binder ratio (S/B), but the rate of increase gradually decreased. When S/B was increased by 0.2, the 7-day compressive strength of three test specimens increased by 6.4%, 5.9%, and 5.7%, respectively, and the 28-day compressive strength increased by 4.3%, 13.2%, and 2.6%, respectively.

Within the range of S/B from 0.8 to 1.4, the enhancement effect of the S/B ratio on the early strength (7-day) showed a decreasing trend, while the improvement in the later strength (28-day) was most significant when S/B was between 1.0 and 1.2. This may be attributed to the synergistic effect of the skeleton effect of the fine aggregate and the binding action of the cement paste within a reasonable range of S/B, which enhances the mortar strength. However, when S/B exceeds 1.2, the suspension state of the fine aggregate in the paste increases, weakening the skeleton effect, so that the strength improvement primarily relies on the increase in cementitious materials, leading to a decrease in the rate of strength increase.

#### 3.1.3. Volume Stability

**Figure 4:**
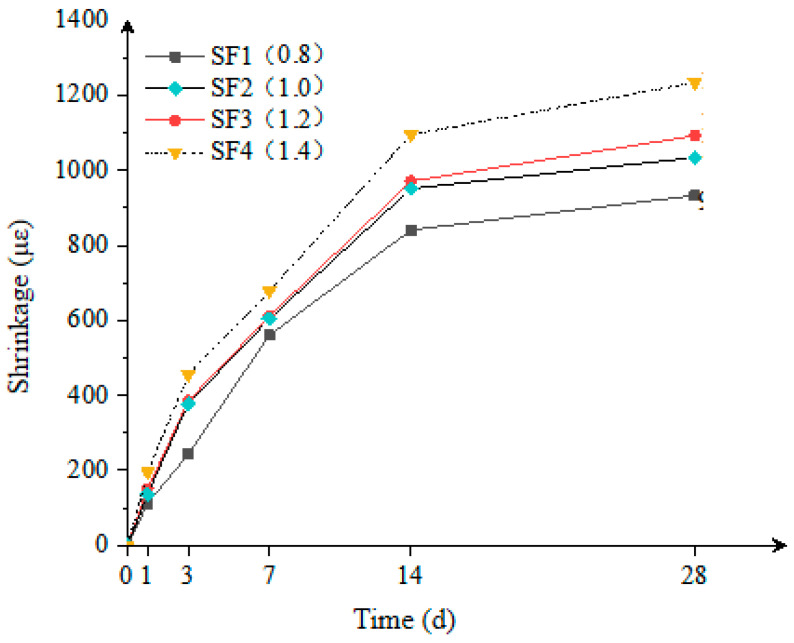
Effect of the sand-to-binder ratio on the shrinkage of the composite mortar.

As shown in Figure 4, within the range of S/B from 0.8 to 1.4, an increase in the sand-to-binder ratio leads to a gradual increase in the volumetric shrinkage of the composite mortar. When S/B was 1.4, the volumetric shrinkage was 13.0% higher compared to when S/B was 1.2, indicating that an excessively high sand-to-binder ratio is detrimental to the volumetric stability of the mortar. Fine aggregates play a skeletal role in the mortar and are a key factor in improving volumetric stability.

As the sand-to-binder ratio increases, the relative content of fine aggregates decreases, weakening their skeletal effect and making the shrinkage characteristics of the mortar more influenced by the cementitious materials. Simultaneously, an increase in the amount of binder paste results in higher autogenous shrinkage and drying shrinkage, further reducing volumetric stability. In PACFST systems (void ratio = 34.7%), the increased cementitious content (605–770 kg/m^3^) elevates shrinkage because the rigid aggregate skeleton provides finite restraint, and additional paste increases the unrestrained shrinkage component.

Therefore, to ensure that the composite mortar possesses both good strength and stability, it is recommended to control the sand-to-binder ratio within the range of 1.0 to 1.2 in order to balance volumetric stability and mechanical properties.

### 3.2. Preparation of Mixes with a Low Water-to-Binder Ratio

#### 3.2.1. Workability

As shown in Table 3, when the water-to-binder ratio (W/B) of the composite mortar was reduced from 0.32 to 0.30, the flow time increased from 27 s to 34 s, indicating a significant decrease in fluidity. However, when W/B was further reduced to 0.28, due to the optimization of admixture dosage and the incorporation of various components such as high-efficiency air-entraining agents and defoamers, uniform, fine, and closed bubbles were formed within the paste. This effectively increased the paste volume, reduced the friction between cementitious material particles, and improved fluidity, maintaining a flow time of 35 s, which was only 1 s longer than that at W/B = 0.30.

In addition, all groups of the composite mortar exhibited low sensitivity to admixture dosage. Once the admixture dosage reached the minimum value required to ensure stable working performance, even if an additional 50% was added, the paste remained stable, without bleeding or segregation. This indicates that the optimized combination of appropriate amounts of admixtures can not only improve the rheological properties of the paste but also ensure its good construction adaptability [42].

#### 3.2.2. Mechanical Properties

**Figure 5:**
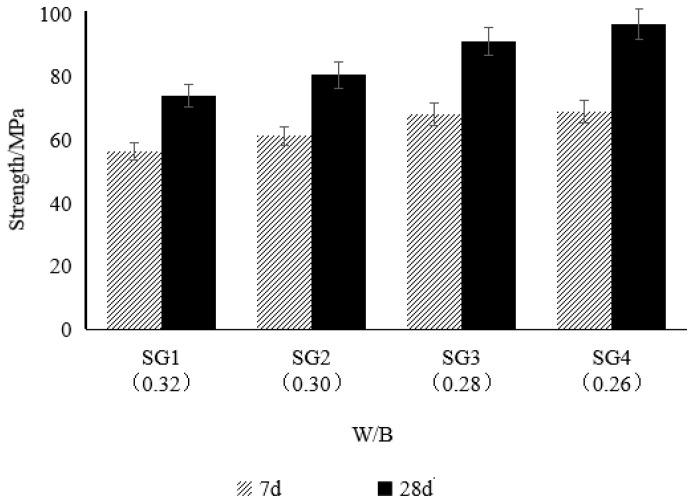
Influence of the low water-to-binder ratio on the strength of the composite mortar.

As shown in Figure 5, the compressive strength of the composite mortar increased as the water-to-binder ratio (W/B) decreased. Within the range of W/B from 0.26 to 0.32, for every 0.02 reduction in W/B, the 7-day strength increased by 8.5%, 11.3%, and 1.3%, respectively, and the 28-day strength increased by 8.9%, 13.2%, and 5.9%, respectively. The results indicate that the trend of strength improvement with decreasing W/B is consistent for both the early (7-day) and later (28-day) stages of the composite mortar, with the most significant strength increase observed in the range of W/B from 0.28 to 0.30.

The main reason for the increase in strength with decreasing W/B is that the reduction in mixing water lowers the residual moisture within the hardened paste. During cement hydration, excess water evaporates to form pores, which reduces the density and mechanical properties of the mortar. However, under conditions of a lower W/B, most of the water is consumed by the hydration reaction, reducing the porosity within the paste and improving its density, thereby significantly enhancing the compressive strength [43]. Therefore, reasonably reducing the water-to-binder ratio is an effective strategy to improve the strength of the composite mortar [44].

#### 3.2.3. Volume Stability

**Figure 6:**
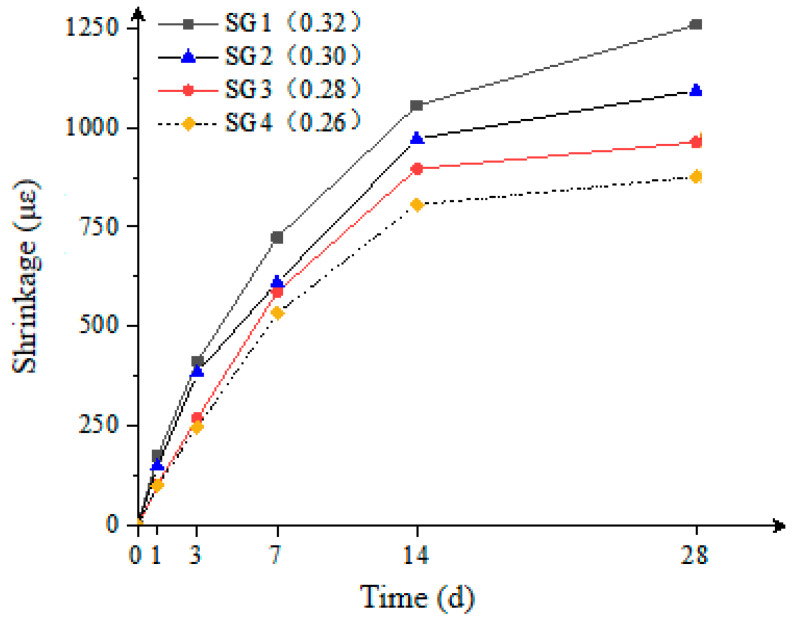
Effect of the water-to-binder ratio on the shrinkage of the composite mortar.

As shown in Figure 6, within the range of W/B = 0.26 to 0.32, the volumetric shrinkage of the composite mortar decreased as the water-to-binder ratio decreased. Specifically, the SG3 group with W/B = 0.28 exhibited a 23.4% reduction in 28-day shrinkage compared to the SG1 group with W/B = 0.32, indicating that appropriately lowering the water-to-binder ratio helps improve volumetric stability. The mechanism by which the water-to-binder ratio affects the volumetric stability of the composite mortar is similar to that of strength changes. At higher water-to-binder ratios, excess water in the paste forms free water droplets after hardening, which evaporate to produce pores, leading to volumetric shrinkage. However, under low water-to-binder ratio conditions, most of the mixing water is consumed by the hydration reaction, reducing the amount of free water and porosity within the paste, thereby effectively minimizing volumetric shrinkage.

Based on previous experimental data, for the composite mortar used in the preparation of preplaced aggregate concrete-filled steel tubes (PACFSTs), the water-to-binder ratio should not be less than 0.28 to ensure both good volumetric stability and workability.

### 3.3. Influence of the Expansive Agent

#### 3.3.1. Workability

As shown in Table 5, the addition of an expansive agent had a minimal impact on the flowability of the composite mortar, with the flow time of each group remaining between 33 s and 36 s. This indicates that under the experimental conditions, the influence of the expansive agent on the rheological properties of the paste was not significant and could be disregarded as a primary consideration. However, the effect of the expansive agent content on the 28-day compressive strength was more pronounced. When the content increased from 0% to 4%, the compressive strength improved from 91.1 MPa to 92.8 MPa. However, as the content continued to increase to 6% and 8%, the strength decreased to 88.3 MPa and 76.3 MPa, respectively. This suggests that an appropriate amount of the expansive agent can compensate for mortar shrinkage and enhance density, whereas excessive addition may lead to an increase in internal porosity, thereby reducing strength.

#### 3.3.2. Mechanical Properties

**Figure 7:**
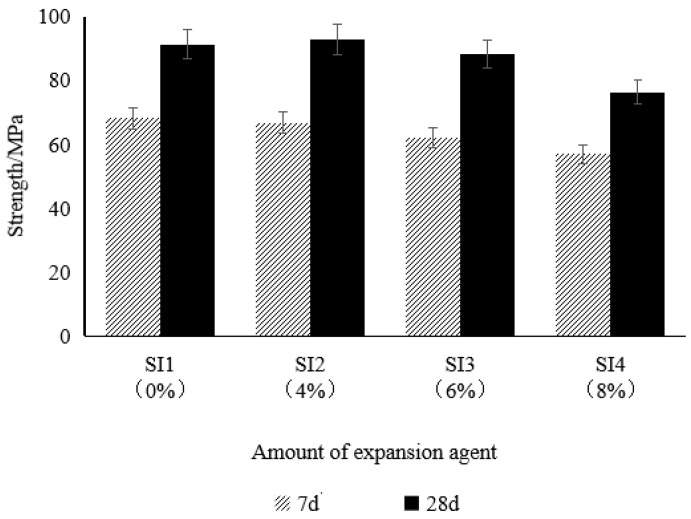
Influence of the expansive agent on the strength of the composite mortar.

As illustrated in Figure 7, the addition of an expansive agent had a certain impact on the compressive strength of the composite mortar. When the expansive agent content was 4% (SI2 group), the 7-day and 28-day strengths showed insignificant changes compared to the control group (SI1). However, when the content increased to 6% (SI3 group), the 7-day and 28-day strengths decreased by 8.7% and 3.1%, respectively. As the content further increased to 8% (SI4 group), the 7-day and 28-day strengths dropped by 16.4% and 16.2%, respectively, indicating that the magnitude of strength reduction significantly increased with the increase in the expansive agent content.

The reason for the strength decrease may be attributed to the relative reduction in the cement content due to the addition of an expansive agent using the external addition method, which results in a decrease in hydration products and subsequently affects the strength development of the mortar [45]. Additionally, the volume effect of the expansive agent may lead to an increase in porosity within the paste, further reducing its mechanical properties. Therefore, to ensure the strength and volumetric stability of the composite mortar, the addition of an expansive agent should be strictly controlled to balance the shrinkage compensation effect and the loss of compressive strength.

#### 3.3.3. Volume Stability

**Figure 8:**
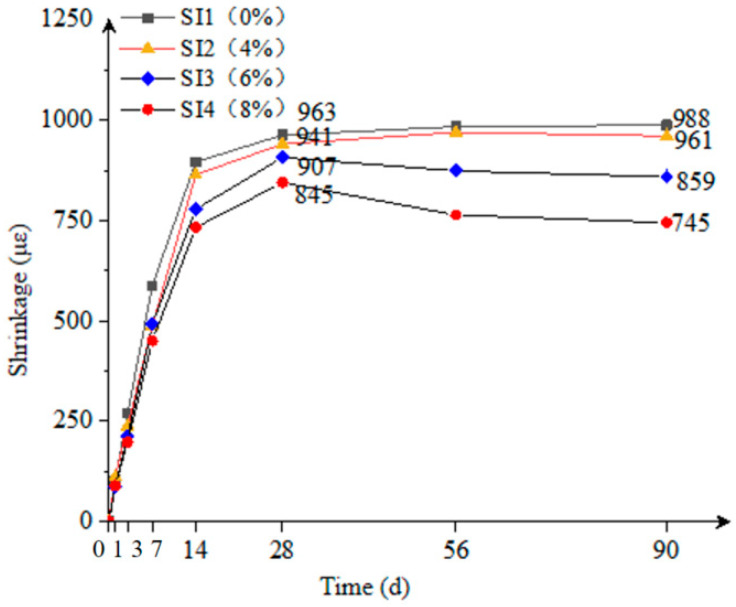
Effect of the expansive agent on the shrinkage of the composite mortar.

As shown in Figure 8, the SI2 group with 4% expansive agent exhibited a reduction in 28-day and 90-day shrinkage of 2.3% and 2.7%, respectively, compared to the control group SI1. The SI3 group with 6% expansive agent showed a decrease in 28-day and 90-day shrinkage of 5.8% and 13.1%, respectively, compared to SI1. The SI4 group with 8% expansive agent demonstrated a significant reduction in 28-day and 90-day shrinkage of 12.3% and 24.6%, respectively, compared to SI1. It is evident that the influence of the expansive agent on the volumetric shrinkage of the composite mortar was primarily manifested in the later stages of shrinkage, as it compensated for the overall shrinkage of the composite mortar after 28 days.

When the expansive agent content was 4%, the effect of compensating for shrinkage was relatively small. As the expansive agent content increased to 6% and 8%, there was a noticeable decrease in both 28-day and 90-day shrinkage of the composite mortar. The expansion mechanism of the expansive agent primarily involved the hydration of MgO and CaO, generating brucite and portlandite, respectively, accompanied by significant volume expansion. Meanwhile, the formation of ettringite (AFt) in the system was attributed to the reaction between gypsum and C_3_A in OPC, which is a typical hydration process independent of the expansive agent. The expansion volume was approximately 2.0 times the original solid volume before hydration.

In the context of this study, it can be concluded that the expansive agent content for the composite mortar used in the preparation of preplaced aggregate concrete-filled steel tubes should be controlled at around 6% to achieve optimal results.

### 3.4. Preparation of Ultra-High-Strength Preplaced Aggregate Concrete-Filled Steel Tubes

The preplaced coarse aggregate gradation of 10~16 mm:16~25 mm = 3:7 (with a void ratio of 34.7%) was selected to prepare the composite mortar preplaced aggregate concrete. Its performance indicators were tested and compared with those of conventional C100 preplaced aggregate concrete-filled steel tubes. The mix proportions are presented in Table 6.

#### 3.4.1. Mechanical Properties

**Figure 9:**
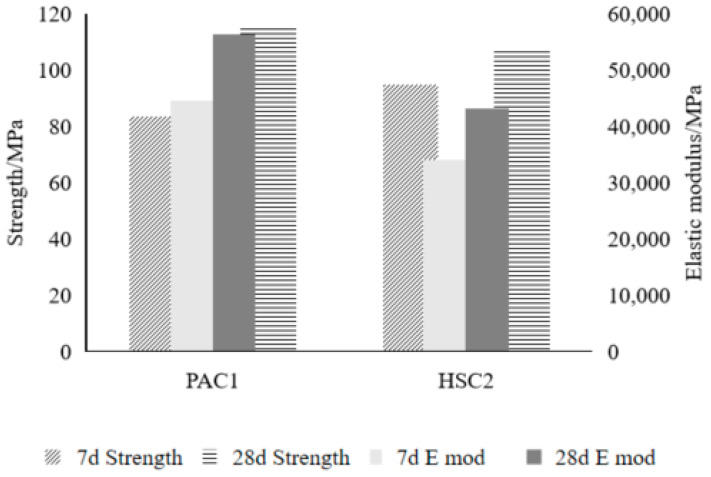
Comparison of mechanical properties.

As shown in Figure 9, the early strength of the PAC1 group was relatively low, with a 7-day strength that was 11.6% lower than that of the HSC2 group. The main reason for this is that the insufficient strength of the paste in the early stages makes it difficult to effectively restrain the displacement and rotation of coarse aggregates under high axial pressure, making concrete members more prone to failure. As the paste strength increases, the bond between the coarse aggregates and the paste strengthens, enhancing the overall load-bearing capacity. The 28-day strength of the PAC1 group was 7.7% higher than that of the HSC2 group, indicating that the optimized composite mortar can effectively improve the later-stage strength performance of PAC.

**Figure 10:**
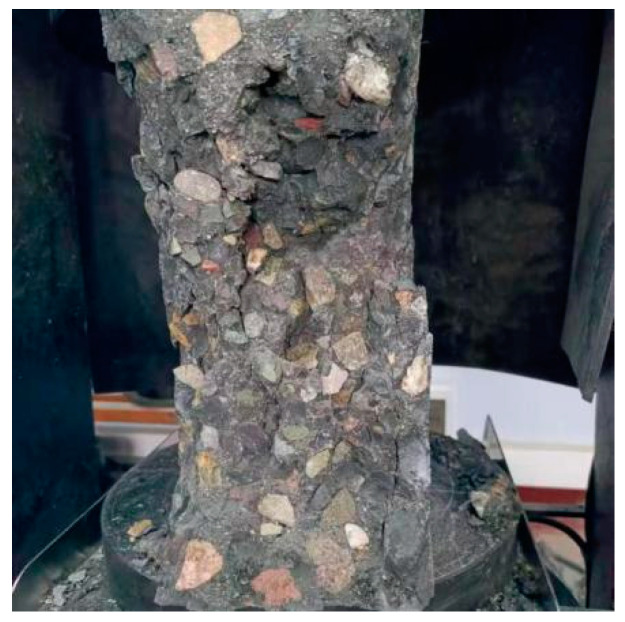
Damage pattern of core concrete with ultra-high-strength composite mortar pre-filled with aggregate.

The failure mode of the composite mortar PAC is shown in Figure 10. It can be observed from the figure that almost all coarse aggregates fractured during compressive failure, indicating that the paste strength was sufficient to effectively restrain the axial displacement and rotation of the coarse aggregates. The interfacial bond strength between the paste and the coarse aggregates was significantly improved, allowing them to form an integrated structure that worked synergistically to bear the axial load and fully utilize the dense skeleton effect of the coarse aggregates. Ultimately, the 28-day compressive strength reached 115.9 MPa.

In terms of the static compressive elastic modulus, the 7-day and 28-day elastic moduli of the PAC1 group were 31.1% and 30.3% higher, respectively, than those of the conventional ultra-high-strength concrete (HSC2 group). This indicates that the aggregate volume content has a significant impact on the elastic modulus of concrete members. A higher coarse aggregate content can increase the stiffness of the concrete, improve the deformation resistance of the members, and enable them to exhibit better elastic performance under high load conditions. While the study demonstrated significant improvements in mortar performance, the critical importance of the steel–concrete bond behavior in PACFST applications requires acknowledgment. Preliminary tests indicated the developed low-shrinkage mortar achieved a 3.2 MPa bond strength, exceeding the Chinese GB 50936-2014 [46] requirements for static loads. However, the preplaced aggregate process may reduce the interface penetration depth by approximately 60% compared to conventional casting. Further research remains necessary to fully characterize bond performance under cyclic loading and long-term exposure, particularly for Eurocode compliance, where mechanical connectors remain essential.

#### 3.4.2. Volume Stability

**Figure 11:**
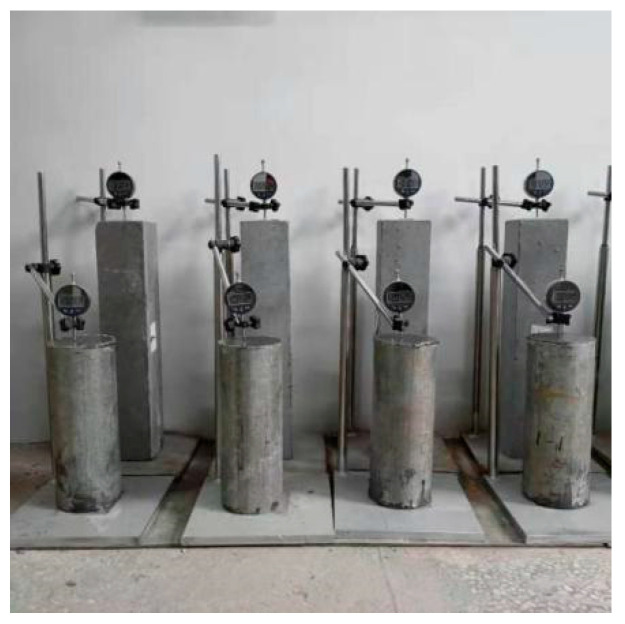
Shrinkage test of preplaced aggregate concrete-filled steel tubes.

**Figure 12:**
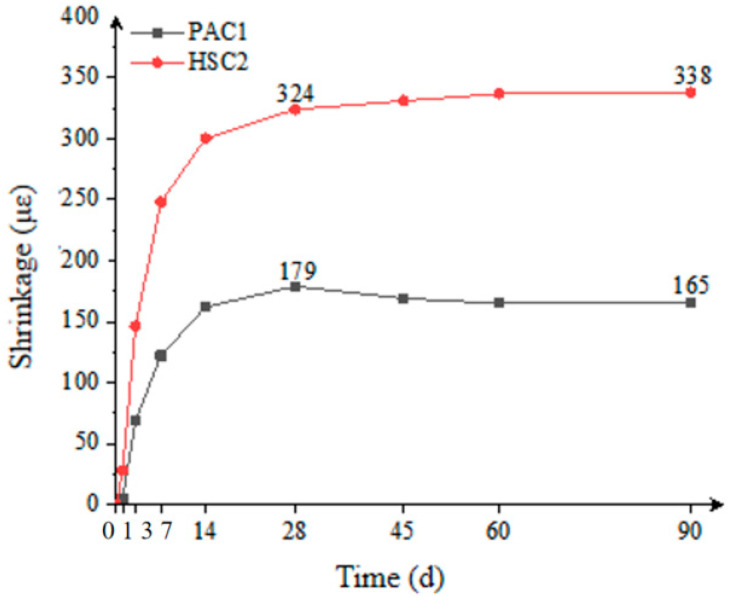
Comparison of the shrinkage performance.

The testing method is illustrated in Figure 11. As shown in Figure 12, the 28-day volume shrinkage of the PAC1 group was 44.8% lower than that of the HSC2 group, and the 90-day volume shrinkage was 51.2% lower. This indicates that preplaced aggregate concrete-filled steel tubes (PACFSTs) exhibit superior long-term volume stability compared to conventional C100 concrete-filled steel tubes (HSC). The main reason for this is that the high volume content of coarse aggregates in the PAC system forms a densely packed structure, which inhibits the volume shrinkage of the composite mortar to some extent, thereby significantly improving the overall volume stability.

Furthermore, during the hydration process, the expansive agent generates expansive hydration products such as brucite and portlandite through the reaction of MgO and CaO. The ettringite (AFt) formation is derived from the sulfate and aluminate phases present in OPC, producing a volume expansion effect. This effectively compensates for the shrinkage of the concrete. Therefore, the ultra-high-strength composite mortar of the PAC1 group performed excellently in reducing volume shrinkage, providing an effective path for optimizing the shrinkage control of preplaced aggregate concrete-filled steel tubes.

## 4. Conclusions

This study aimed to enhance the strength and volumetric stability of preplaced aggregate concrete-filled steel tubes (PACFSTs) by developing a high-performance composite mortar with a low water-to-binder ratio. The main conclusions are as follows:

(1) The influence of the sand-to-binder ratio on the fluidity of the composite mortar is more significant when the ratio is relatively small. However, when the sand-to-binder ratio becomes excessively large, the fluidity no longer improves, and the paste strength decreases.
(2) Based on an appropriate amount of mineral admixtures, by optimizing the sand-to-binder ratio to 1.2, reducing the water-to-binder ratio to 0.28, and supplementing with 6% expansive agent to compensate for shrinkage, a high-performance composite mortar can be produced. Its 28-day compressive strength can reach 88.3 MPa or higher, while also exhibiting excellent fluidity and volume stability.
(3) Using a high-performance composite mortar as the paste material, ultra-high-strength preplaced aggregate concrete-filled steel tubes (PACFSTs) can be prepared. Compared to conventional C100 concrete-filled steel tubes, this PAC system has higher strength and elastic modulus, reduced volume shrinkage, and offers good economic benefits and environmental adaptability. It is suitable for engineering fields such as bridges, tunnels, and high-rise buildings.
(4) While this study demonstrates significant improvements in the mechanical performance and shrinkage resistance, further research should focus on long-term durability under aggressive environmental conditions (e.g., marine exposure, freeze–thaw cycles) and full-scale validation to assess thermal cracking risks and constructability. Additionally, lifecycle cost analysis and carbon footprint assessment are needed to evaluate the economic and environmental viability of this system for large-scale applications.

## Figures and Tables

**Table 1:** Technical indices of the cement.

Strength Class	Specific Surface Area, m^2^·kg^−1^	Flexural Strength, MPa		Compressive Strength, MPa	
		3 d	28 d	3 d	28 d
P·O 42.5	320	5.9	5.4	28.6	46.1
P·O 52.5	364	7.2	10.6	35.1	57.1

**Table 2:** Technical index of the expansive agent.

Setting Time, min		Restricted Expansion Rate, %		Compressive Strength, MPa	
Initial Setting	Final Setting	7 d in Water	28 d in Air	7 d	28 d
168	215	0.026	0.012	29.3	41.6

**Table 3:** Mix design of mortar with different SF contents.

ID	S/B	Cementitious Materials, kg·m^−3^						Flowability	Mechanical Property	
		Cement	FA	SF	Sand	Water	Expansive Agent	Efflux Time	7 d Compressive Strength, MPa	28 d Compressive Strength, MPa
SF1	0.8	605	170	70	1055	317	9	38 s	54.3	68.2
SF2	1	670	190	75	935	281	9	29 s	57.8	71.1
SF3	1.2	730	200	80	840	303	10	27 s	61.2	80.5
SF4	1.4	770	210	85	760	320	10	27 s	64.7	82.6

**Table 4:** Mix ratio of the composite mortar with a low water-to-binder ratio.

ID	S/B	Design Weight	Cementitious Materials, kg·m^−3^						Flowability	Mechanical Property	
			Cement	FA	SF	Sand	Water	WRA	Efflux Time	7 d Compressive Strength, MPa	28 d Compressive Strength, MPa
SG1	0.32	2150	720	200	80	830	320	10	27 s	56.4	73.9
SG2	0.30	2150	730	200	80	840	303	10	34 s	61.2	80.5
SG3	0.28	2250	770	215	85	890	276	16	35 s	68.1	91.1
SG4	0.26	2250	770	215	90	895	280	22	45 s	69	96.5

**Table 5:** Effect of the expansive agent on the properties of the composite mortar.

ID	Expansive Agent	Cementitious Materials, kg·m^−3^							Flowability	Mechanical Property	
		Cement	FA	SF	Sand	Expansive Agent	Water	WRA	Efflux Time	7 d Compressive Strength, MPa	28 d Compressive Strength, MPa
SI1	0%	770	215	85	890	0	276	16	35 s	68.1	91.1
SI2	4%	770	215	85	890	45	276	22	36 s	66.7	92.8
SI3	6%	770	215	85	890	65	276	28	33 s	62.2	88.3
SI4	8%	770	215	85	890	85	264	32	35 s	56.9	76.3

**Table 6:** Concrete mix.

Class	ID	Mix Design, kg·m^−3^							
		Cement	FA	SF	Expansive Agent	Water	Sand	Stone	WRA
C100 PACFST	PAC1	267	75	30	23	96	309	1737	7.6
C100 CFST	HSC2	480	100	90	0	126	704	1013	16.8

## Data Availability

The original contributions presented in this study are included in the article. Further inquiries can be directed to the corresponding author.
